# Methodological challenges and biases in the field of cognitive function among patients with chronic kidney disease

**DOI:** 10.3389/fmed.2023.1215583

**Published:** 2023-08-09

**Authors:** Konstantinos Giannakou, Aleksandra Golenia, Sophie Liabeuf, Jolanta Malyszko, Francesco Mattace-Raso, Ana Farinha, Goce Spasovski, Gaye Hafez, Andrzej Wiecek, Giovanna Capolongo, Giovambattista Capasso, Ziad A. Massy, Marion Pépin

**Affiliations:** ^1^Department of Health Sciences, School of Sciences, European University Cyprus, Nicosia, Cyprus; ^2^Department of Neurology, Medical University of Warsaw, Warsaw, Poland; ^3^Pharmacoepidemiology Unit, Department of Clinical Pharmacology, Amiens University Medical Center, Amiens, France; ^4^MP3CV Laboratory, EA7517, Jules Verne University of Picardie, Amiens, France; ^5^Department of Nephrology, Dialysis and Internal Medicine, Medical University of Warsaw, Warsaw, Poland; ^6^Department of Geriatric Medicine, Erasmus MC University Medical Center, Rotterdam, Netherlands; ^7^Department of Nephrology, Hospital de Vila Franca de Xira, Vila Franca de Xira, Portugal; ^8^University Department of Nephrology, Clinical Centre “Mother Theresa”University Sts Cyril and Methodius, Skopje, North Macedonia; ^9^Department of Pharmacology, Faculty of Pharmacy, Altinbas University, Istanbul, Türkiye; ^10^Department of Nephrology, Transplantation and Internal Medicine, Medical University of Silesia in Katowice, Katowice, Poland; ^11^Department of Translational Medical Sciences, University of Campania "Luigi Vanvitelli", Naples, Italy; ^12^Biogem Research Institute, Ariano Irpino, Italy; ^13^Service de Néphrologie, CHU Ambroise Paré, Assistance Publique - Hôpitaux de Paris & Université Paris-Saclay (Versailles-Saint-Quentin-en-Yvelines), Boulogne Billancourt, France; ^14^Inserm U-1018 Centre de Recherche en Épidémiologie et Santé des Populations (CESP), Équipe 5, Paris-Saclay University, Versailles Saint-Quentin-en-Yvelines University, Villejuif, France; ^15^Departement of Geriatric Medicine, Ambroise Paré Hospital, AP-HP, Boulogne-Billancourt, France

**Keywords:** chronic kidney disease, CKD, cognitive function, cognitive impairment, biases, methodological challenges

## Abstract

Chronic kidney disease (CKD) affects approximately 850 million people globally and is associated with an increased risk of cognitive impairment. The prevalence of cognitive impairment among CKD patients ranges from 30 to 60%, and the link between CKD and cognitive impairment is partially understood. Methodological challenges and biases in studying cognitive function in CKD patients need to be addressed to improve diagnosis, treatment, and management of cognitive impairment in this population. Here, we review the methodological challenges and study design issues, including observational studies’ limitations, internal validity, and different types of bias that can impact the validity of research findings. Understanding the unique challenges and biases associated with studying cognitive function in CKD patients can help to identify potential sources of error and improve the quality of future research, leading to more accurate diagnoses and better treatment plans for CKD patients.

## Introduction

Chronic kidney disease (CKD) is a non-communicable disease with a global prevalence of 9%, corresponding to roughly 850 million cases (1). CKD is an independent risk factor associated with the development of cognitive impairment (2–4). The prevalence of cognitive impairment among patients with CKD ranges from 30 to 60%, depending on the definition and assessment of cognitive impairment and on the stage of CKD. In comparison to general population, CKD patients, especially those in end-stage-kidney-disease (ESKD), present a 3–4-fold higher frequency of lower cognitive function (5–7). Cognitive changes can manifest at an early stage of CKD (5, 8). Traditional vascular risk factors do not fully account for the high prevalence of cognitive impairment in patients with CKD. Non-traditional vascular risk factors such as inflammation, anemia, endothelial dysfunction, increasing age and dialysis vintage, or classic and novel electrolyte abnormalities (especially hyponatremia and hyperphosphatemia) or uremic toxins accumulation, are common among patients with CKD and frequently present as contributing risk factors for cognitive impairment (9–11). However, the link between CKD and cognitive impairment is not completely understood, promoting research in this specific area (12).

Early stages of CKD are highly prevalent in the general population and are already known to be associated with cognitive dysfunction (13). Indeed, epidemiological data suggest that individuals at all stages of CKD have a higher risk of developing cognitive disorders and dementia (14). Different nonpharmacological and pharmacological approaches can be employed to mitigate cognitive impairment and reduce its impact on the daily lives of individuals with CKD (15). It is essential to review the methodological challenges and biases in the field of cognitive function among patients with CKD because accurate and reliable research is crucial for improving the diagnosis, treatment, and management of cognitive impairment in this population. Understanding the unique challenges and biases associated with studying cognitive function in CKD patients can help to identify potential sources of error and improve the quality of future research. Furthermore, providing solutions to these challenges can help to develop better methods of assessing cognitive function in CKD patients, leading to more accurate diagnoses and better treatment plans. Ultimately, improving our understanding of the relationship between CKD and cognitive function can help to improve the quality of life and long-term outcomes for CKD patients. Therefore, the aim of this narrative review is to explore the unique challenges associated with studying cognitive function in CKD patients, including conceptual concerns and methodological challenges, difficulties in defining exposure indicators and outcomes and assessing cognitive function, as well as to identify potential solutions to address these challenges.

## Methodological challenges and study design issues

**Figure 1:**
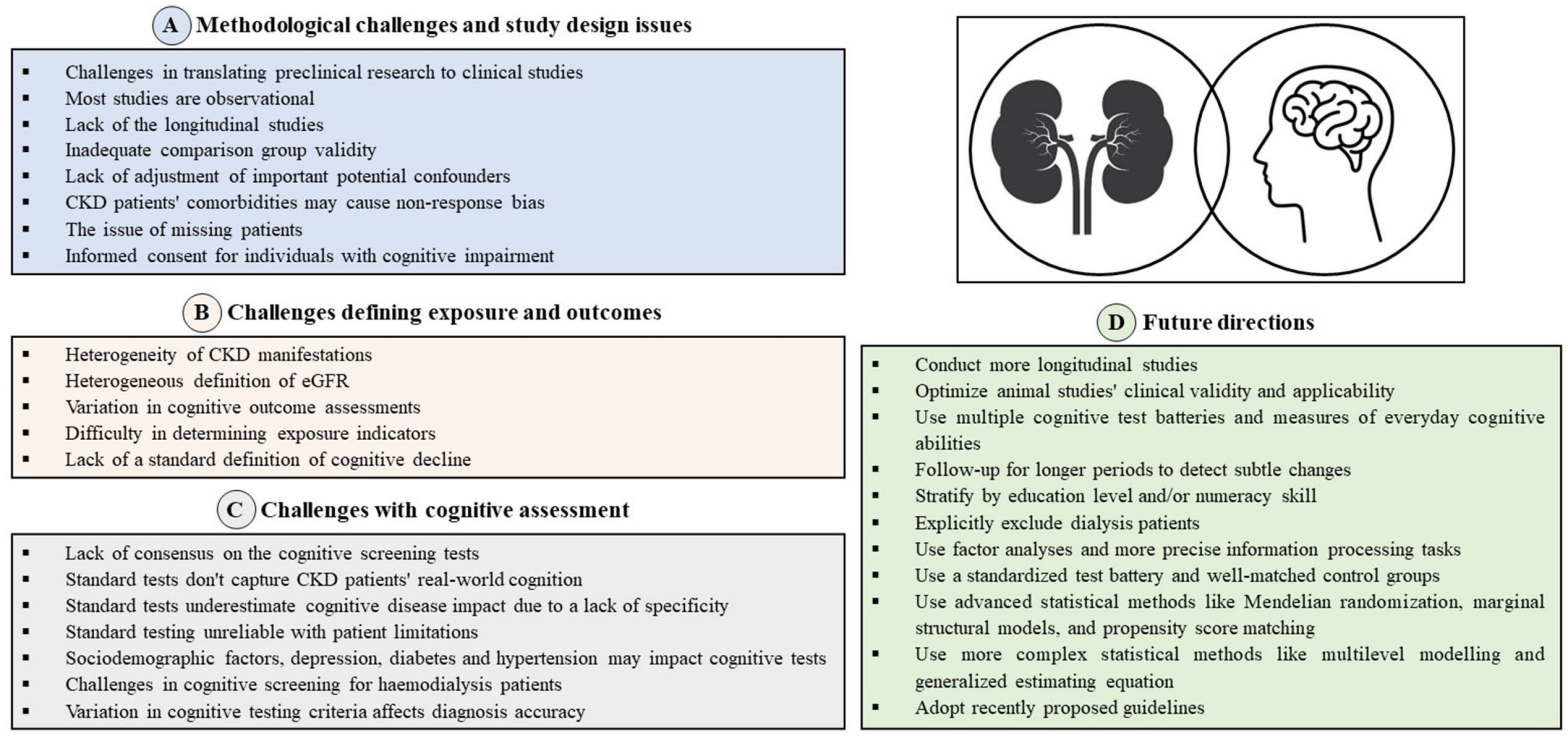
**(A)** Methodological challenges and study design issues. **(B)** Challenges defining exposure and outcomes. **(C)** Challenges with cognitive assessment. **(D)** Future direction.

During the assessment of cognitive function in CKD patients, it is essential to consider the preclinical data available from animal studies. The results from these studies provide important insights into the cognitive impairment and behavioral changes that may occur in CKD. However, it is crucial to acknowledge that animal studies have many limitations, including small sample sizes, lack of randomization and blinding, and most importantly anatomical and physiological differences between animals and humans (16). Therefore, the findings of animal studies should be interpreted with caution, and further investigations such as human clinical trials are necessary to confirm the validity and applicability of these results in the clinical setting. Nonetheless, despite these limitations, preclinical animal studies remain a crucial tool for advancing scientific knowledge and developing new therapies for various diseases, including CKD-associated cognitive impairment (Figure 1A).

The majority of studies examining cognitive function in patients with CKD are observational in nature, with a significant proportion being cross-sectional studies, which presents a further limitation (4, 5). Cross-sectional studies assess both the outcome and exposure at the same time, making it challenging to establish a causal relationship between them. Moreover, these studies often use single measures to evaluate cognitive function, failing to account changes in cognitive function over time, leading to a less comprehensive understanding of the disease’s progression.

When considering the internal validity, different types of bias are frequent. According to previous systematic reviews and meta-analyses, the quality of primary studies in this area was generally low to medium as the majority contain methodological limitations (4, 5, 17, 18). For instance, the studies included in these reviews were primarily found to have inadequate comparison group validity, indicating that the groups being compared were not adequately matched for important demographic and health status variables. Only a small number of studies were found to be well-matched in these areas, indicating that the overall quality of evidence may be limited (5). Earlier systematic reviews and meta-analyses that combined findings from observational studies investigating cognitive function in individuals with CKD, including those undergoing peritoneal dialysis or with ESKD, have found that some studies included strategies to address common confounding factors, such as age, sex, health-related factors, and cardiovascular disease (CVD) risk factors, either by excluding them or adjusting for them (4, 5, 17, 18). However, they also found that many studies did not take into account significant confounders such as education, physical activity, or depression (4, 5, 17, 18). Various other potential confounders still exist that are associated with psychosocial and treatment factors such as depression, polypharmacy, and malnutrition, biological intrinsic factors such as subclinical atherosclerosis, anemia, cortical atrophy of the brain, and hyperparathyroidism, and dialysis-related factors such as chronic cerebral micro-embolism, lacunar infarcts, and microbleeds (19).

Selection bias is a significant topic in research on cognitive impairment due to factors such as non-random enrolment, refusal to participate, differential survival, and differential attrition of enrolled participants. These processes can bias effect estimates. Non-response bias and missing cases are also common issues that can significantly impact the validity of research findings. In studies of cognitive function among CKD patients, non-response bias is particularly relevant, as CKD patients may be reluctant or unable to participate due to comorbidities, fatigue, or other symptoms. Patients with CKD, including those on dialysis, often experience limitations that can affect their cognitive function testing. These limitations include advanced age, diabetic complications, arterio-venous fistula for dialysis or paresis, pain, lack of motivation due to depression, impaired vision, and motor difficulties. Standard cognitive function testing, such as the Mini-Mental State Examination (MMSE), is only valid for patients who are visually, physically, and motivationally able to participate, which raises concerns about the accuracy of measuring cognitive function in CKD patients. As a consequence, eligible patients with high cognitive performance may be excluded if they have visual or motor impairments (20). Earlier research has suggested that excluding individuals with CKD based on extensive criteria and inconsistent use of exclusion thresholds such as MMSE scores <24 or diagnosed with dementia, can lead to an imprecise evaluation of their cognitive function (6, 20–22). Excluding patients with cognitive impairment could potentially overestimate cognitive function, which implies that existing studies about cognitive function in CKD are of limited generalizability to the CKD population. However, the impact of other selection mechanisms, such as visual, motivation, motor, and language impairments, on cognitive function testing remains uncertain and could result in both over- and underestimation.

In clinical studies, the process of informed consent requires individuals who have the capacity to understand the research protocol to decide whether they voluntarily agree to participate in a study. Institutional Review Boards (IRBs) have strict requirements for the informed consent process that include both verbal and written descriptions of what participation in the study would entail, including the purpose of the research and any potential risks and benefits. Studies involving people with impairment of cognitive function are subject to vigilance by IRBs as a vulnerable population. Potential ethical issues with research involving people with cognitive disorders include respecting their abilities to make decisions, securing consent to participate from proxy decision-makers (e.g., family members) or simply excluding this population from the study because of cognitive impairment.

## Challenges defining exposure and outcomes

The first difficulty in defining exposure indicators and outcomes in the field of cognitive function among CKD patients is the fact that CKD is a heterogeneous condition that can manifest in different ways (decreased GFR, albuminuria), with various causes, stages (degrees of severity) and interventions (conservative treatment, dialysis, transplantation). Moreover, the use of traditional creatinine-based formulae to estimate eGFR was inferior to the eGFR cystatin C formula, which proves to be the best eGFR for assessing associations with CVD and mortality (23). In addition, current CKD studies investigate the association between CKD and cognitive decline using cognitive outcomes that assess different abilities to differentiate their relationship with the disease (4). The outcome variables can be dichotomous, such as dementia, impairment, or deficit; ordinal categories of performance level; or continuously distributed test scores that represent performance level (19). The term cognitive impairment may be used when cognitive status is established by clinical criteria, while the comparative term “deficit” indicates a lower average level of performance compared to reference groups. Longitudinal changes in performance may be characterized as a decline. This primary difficulty variability can affect the cognitive function of patients differently and make it challenging to establish a standard definition of CKD-related cognitive impairment. Determining the appropriate indicators of exposure may be most difficult in studies of cognitive function among CKD patients. Researchers must consider various factors that may affect cognitive function, such as the duration and severity of CKD, comorbidities (e.g., depression, hypertension, diabetes, CVD), and treatment modalities (dialysis, transplantation) (Figure 1B).

## Challenges with cognitive assessment

Cognitive functions may decline in the course of CKD (24). It seems that the prevalence of cognitive decline, including Mild Cognitive Impairment (MCI) and dementia, in patients with ESKD and undergoing renal replacement therapy (RRT) may be underdiagnosed. Therefore, more attention should be paid to screening for cognitive functions in CKD patients. Cognitive assessment may be performed at varying levels of specialist care and for several reasons, such as screening for early diagnosis of cognitive impairment, classifying causes of cognitive problems, monitoring disease progression and severity or treatment outcomes (25, 26) (Figure 1C).

The assessment of cognitive function in CKD patients seems to be difficult for various reasons. Various cognitive screening tools are used in everyday clinical practice to assess cognitive function (27, 28). The cognitive tests vary in sensitivity and specificity, and may not be sensitive enough to detect subtle changes cognitive ability, especially in patients with MCI (27, 28). There is still no consensus on which test is better, and the choice depends on the clinician’s familiarity with the test, the purpose of the cognitive assessment, translation availability, copyright, test simplicity, duration, and its reliability and validity (29). The usefulness of screening tests for identifying cognitive impairment should have a good sensitivity above 80% and an acceptable specificity above 60% (30). In addition, standard cognitive screening tests may not be able to distinguish between specific aspects of cognition that are most impacted and often fail to consider the various real-world demands that patients with CKD may face. Cognition comprises various discrete domains, covering a wide range of processes, such as visuo-spatial perception, auditory and visual memory, attention span, motor function, and mathematical reasoning (5). For instance, some brief cognitive screening tools, such as the MMSE, which focus on the cortical cognitive domain, particularly memory, and were developed to detect dementia in other conditions, including Alzheimer’s disease, may be less sensitive in identifying cognitive impairment in patients with CKD. Like the MMSE, the Modified Mini-Mental State Examination (3MS) and the 6-item-Screener tests can only indicate cognitive impairment at a broad, global level, which lacks specificity and does not provide useful information to healthcare providers who may need to focus their attention on specific areas. Thus, because these tests may not be sensitive enough to detect subtle cognitive changes, relying solely on standardized cognitive tests may not provide a complete picture of the cognitive functioning of CKD patients, potentially leading to an underestimation of the disease’s true impact. In addition, the reliability of standard testing, such as the MMSE and other written assessments, is questionable when administering them to patients who may have visual, physical, or motivational limitations. Due to the varied restrictions that patients on dialysis often experience, it is uncertain if their cognitive function can be accurately measured without any prejudice. Also, some more detailed cognitive testing cannot be done if there is severe cognitive impairment. Unfortunately, only one study has validated various cognitive screening tests as a diagnostic tool among CKD patients (31). Likewise, the use of different criteria to assess cognitive performance may result in different estimates of cognitive impairment prevalence, making it difficult to accurately diagnose cognitive deficits.

Additionally, various factors, such as physical disability (hearing and visual impairment, paresis of the limbs) and pre-existing severe cognitive impairment, which make it impossible to conduct cognitive evaluations. These factors often result in the exclusion of CKD patients from cognitive testing, further complicating the validation of cognitive tests in this patient group (20). Furthermore, socio-demographic variables, e.g., sex, age, and educational level may also influence total cognitive performance as well as single cognitive domains (32). It is also well known that depression is a recognized risk factor for cognitive dysfunction, especially affecting memory, attention, and psychomotor speed, in the general population (33), and this should be considered when assessing cognitive functioning (34–36). This association is particularly significant in the geriatric population, where distinguishing between depression and MCI can be challenging (37). The signs and symptoms of both conditions may overlap, and an accurate diagnosis requires neurophysiological evaluation (38, 39). Evidence suggests that depression may coexist with MCI and even progress to dementia, indicating a more common phenomenon rather than two distinct conditions (40). While the term “pseudodementia” has been proposed for these cases, its use remains controversial (41). In some cases, an antidepressant treatment may be necessary to evaluate possible improvement in the cognitive function. Depression is a prevalent comorbidity in patients with CKD, with a prevalence of 30% (42), which is much higher than in the general population. Moreover, depression is now recognized as a risk factor for cognitive impairment in CKD patients (43), although data on the co-occurrence of both conditions are limited. Likewise, patients who have CKD also commonly suffer from hypertension (44), which could potentially impact the outcome of cognitive tests. This is because hypertension tends to impact the frontal and subcortical regions of the brain the most, which are responsible for executive function and information processing (45, 46). Thus, it is worth considering the implementation of screening examinations for depression and hypertension as part of routine clinical care for patients with CKD.

Furthermore, cognitive evaluation should be considered in all patients after the resolution of a delirium episode. Delirium is common in patients with CKD, especially those undergoing RRT (47), and is a strong predictor of new-onset dementia and acceleration of existing cognitive decline (48). It appears that, in some patients with CKD, delirium may be the first symptom of cognitive dysfunction. Moreover, hemodialysis patients present a challenge for inclusion in studies utilizing cognitive screening tools. This challenge stems from the difficulty in timing patient interviews, as it is not feasible to administer the screening tests during or immediately after hemodialysis session, as patients may experience fatigue. Requesting patients to attend the clinic on a non-dialysis day for screening tests may also pose a challenge, as patients may be unwilling to comply.

Cognitive ability is typically assessed in subjects from heterogeneous background using a battery of cognitive tests covering different cognitive domains (49). The use of different criteria to assess and integrate cognitive performance in a neurocognitive battery influences the estimated prevalence of cognitive impairment. Experts in cognition propose a framework for optimal diagnosis accuracy, including 3 phases: determination of cut-off scores, determination of the optimal combination of scores, integration of multiple cognitive scores (49). This strategy seems more effective than the determination of the optimal battery for the diagnosis of a given disease which would not be meaningful in CKD patients. Indeed, even if we suppose that CKD patients may exhibit a specific pattern of cognitive impairment, neurocognitive disorders from another etiology may coexist with CKD, especially in older patients (50).

## Conclusion and future directions

This review delves into the primary obstacles linked to studying cognitive function in CKD patients, encompassing conceptual issues, methodological hurdles, and challenges in defining exposure indicators and outcomes, as well as evaluating cognitive function (Figure 1D). There is significant potential for further research to explore in the field of cognitive function among patients with CKD. There is a pressing need for more longitudinal studies that can capture changes in cognitive function over time, allowing for a more accurate characterization of the disease’s trajectory and providing more robust insights into potential interventions to improve cognitive function in CKD patients. As we have mentioned above, the results from animal studies examining behavior and cognition need to be optimized and their clinical validity and applicability should be carefully evaluated. To improve the methodological quality of studies in this area, it is recommended that researchers conduct more studies using multiple cognitive test batteries and measures of everyday cognitive abilities that are relevant to patients’ understanding of their disease and treatment. This will enable researchers to obtain a comprehensive understanding of cognitive function in CKD patients. Longer longitudinal follow-up periods will allow for the detection of subtle changes in cognitive function over time and assess the impact of different CKD stages on cognitive function. Stratification by education level and/or numeracy skill can help control potential confounding factors that may influence cognitive function. Additionally, explicitly excluding dialysis patients could be beneficial to isolate the effects of CKD itself on cognitive function, as chronic dialysis treatment can significantly impact cognitive function. Furthermore, factor analyses that identify theoretically relevant cognitive domains and the use of more precise information processing tasks are recommended. A consensus on a standardized test battery would make comparisons of studies and subgroups clearer, while well-matched CKD and control groups can minimize the impact of confounding variables and increase the validity of the study findings. Moreover, using more advanced statistical methods, such as Mendelian randomization, marginal structural models, and propensity calibration, can help better control for potential confounding variables and improve the accuracy of the results. Similarly, more complex statistical methods like multilevel modeling and generalized estimating equation can help account for the hierarchical nature of data and improve the precision of the estimates. These methods can provide a more rigorous analysis and better insight into the associations between CKD and cognitive function. Lastly, insufficient reporting has made assessing articles challenging in some cases, and adopting recently proposed guidelines can help alleviate this issue for advancing the field.

## Author contributions

KG conceived and wrote the majority of the manuscript. AG, SL, JM, FM-R, AF, GS, and MP contributed to writing parts of the manuscript. GH, AW, GCapo, ZM, and GCapa critically revised the manuscript. All authors reviewed and approved the manuscript for publication.

## Funding

This article/publication was based upon work from COST Action CA19127-Cognitive Decline in Nephro-Neurology: European Cooperative Target (CONNECT), supported by COST (European Cooperation in Science and Technology), www.cost.eu. COST (European Cooperation in Science and Technology) is a funding agency for research and innovation networks. Our Actions help connect research initiatives across Europe and enable scientists to grow their ideas by sharing them with their peers. This boosts their research, career, and innovation.

## Conflict of interest

The authors declare that the research was conducted in the absence of any commercial or financial relationships that could be construed as a potential conflict of interest.

## Publisher’s note

All claims expressed in this article are solely those of the authors and do not necessarily represent those of their affiliated organizations, or those of the publisher, the editors and the reviewers. Any product that may be evaluated in this article, or claim that may be made by its manufacturer, is not guaranteed or endorsed by the publisher.
